# The Role of Cyanobacterial External Layers in Mass Transfer: Evidence from Temperature Shock Experiments by Noninvasive Microtest Technology

**DOI:** 10.3390/microorganisms8060861

**Published:** 2020-06-07

**Authors:** Yan Xiao, Lingxin Liu, Zhe Li, Yuran Cheng

**Affiliations:** CAS Key Laboratory on Reservoir Water Environment, Chongqing Institute of Green and Intelligent Technology, Chinese Academy of Sciences; No. 266 Fangzheng Avenue, Shuitu Hi-tech Industrial Park, Shuitu Town, Beibei District, Chongqing 400714, China; yxiao@cigit.ac.cn (Y.X.); liulingxin17@mails.ucas.ac.cn (L.L.); chengyuran18@mails.ucas.ac.cn (Y.C.)

**Keywords:** cyanobacteria, external layers, mass transfer, temperature shock, noninvasive microtest technology (NMT)

## Abstract

Groundwork on cyanobacterial external layers is crucial for an improved understanding of the persistent dominance of cyanobacteria in freshwaters. In this study, the role of two morphotypes of external layers in *Microcystis* and *Nostoc* in mass transfer and instantaneous temperature shock were explored by noninvasive microtest technology (NMT) after a series of pretreatments, to obtain the external layers retained or stripped samples. The results showed no statistical influence on photosynthetic activity between retained and stripped samples in both *Microcystis* and *Nostoc*. External-layer-retaining strains had higher net O_2_ effluxes than stripped strains. Moreover, the net NH_4_^+^ influx was significantly higher for the sheath retaining *Nostoc* than for the stripped sample, indicating that external layers might be an important feature driving mass transfer in cyanobacteria. However, the role of slime in NH_4_^+^ absorption was limited compared with that of sheath. In addition, external-layer-retaining strains exhibited a longer response time to instantaneous temperature shock, greater net O_2_ effluxes at a 4 °C shock and lower net O_2_ influx at a 35 °C shock, which were interpreted as reflecting a tolerance to temperature fluctuation over short time scales via a buffer function of external layers to stabilize cell activity, ameliorating the efficiency of photosynthesis and respiration. These results advance current knowledge regarding the external layers, especially the dense sheath, involved in the mass transfer in cyanobacteria, and provide new clues concerning the adaptive strategies of cyanobacteria under global climate changes.

## 1. Introduction

Surface coats are widespread, surrounding microorganisms as well as some plant and animal cells. By comparing the surface coats of a variety of cell types, Bennett [1] proposed a generalized terminology of “glycocalyx” for this biochemical structure. Over the past decades, this structure has received increasing attention; yet, the terminology is often confused and not strictly followed. Initially, it was referred to as the sheath and/or capsule based on morphological aspects in microorganisms [2,3]. The terms mucilage and slime were also used in some earlier publications [4,5]. Later, because the microbial surface layer is mainly composed of carbohydrates and can be secreted outside the cell, it was termed the capsular polysaccharides (CPS) [6]. In recent years, some researchers have further divided this structure into loosely bound exopolysaccharides (LB-EPS) and tightly bound exopolysaccharides (TB-EPS) in terms of binding to cells [7,8]. Instead, regardless of the differences in the definitions above, herein, we tend to use a more generalized term, “external layers”, focusing on the surface coats that perform specific eco-physiological functions to support cyanobacteria in the natural environment.

Cyanobacteria possess an outermost structure around their cells, filaments or colonies, which differs from that observed in the bacterial group [9]. The appearance of the external layers in cyanobacteria exhibits a greater variety in terms of form, structure and complex morphology [10]. Rossi and De Philippis [11] indicated that these external layers of cyanobacteria comprise of sheath, capsule and slime, which differ in thickness, consistency and appearance. During the life cycle of cyanobacteria, some of these layers can release polysaccharides (RPS) into the surrounding, causing an increase in viscosity of the culture medium [12]. Generally, the major changes in these external layer types are closely related to the eco-physiological status of the cyanobacteria. Nevertheless, the current fragmented information is unable to support further elucidation of the functions of external layers, and a clearer definition with scientific consensus could not be found.

The existence of external layers (sheath, capsule and slime) in cyanobacteria is generally considered necessary to infer its adaptive strategies in the natural environment [13]. When these layers are present in cyanobacteria, they are likely to perform a protective role against ultraviolet radiation, oxidation, toxic compounds, desiccation and predation by protozoans [14]. More specifically, studies have shown that these external layers are essential for maintaining the colonial morphology of cyanobacteria, and they participate in a wide array of physiological processes, including cell-to-cell interactions, adhesion and aggregation, which further influence the fate of blooms [15]. Cyanobacterial external layers exhibit some typical characteristics that provide a competitive advantage over other competitors throughout its life cycle.

Current knowledge has shown that the combination of climate change, nutrient enrichment and cellular eco-physiological traits favor the dominance of cyanobacteria [16]. Cyanobacteria, considering *Microcystis* as an example, always occur in aggregates or colonies under natural conditions, but they tend to exist as single cells or a few paired cells in laboratory pure cultures [17]. In general, cyanobacteria grow much better in natural waters than in the laboratory under the same concentrations of inorganic nutrients [18]. In this context, laboratory species are regarded as debilitated and semisick cells, due to the morphological change [19]. A different physiological microenvironment has been described between colonies and dispersed cyanobacterial cells [20]. The formation and retention of external layers provides a physical barrier between cyanobacterial cells and their adjacent environment, simultaneously providing a microenvironment around the cells, wherein essential nutrients and trace elements are concentrated [21,22]. In addition, the unique eco-physiological traits, especially the morphology of this outermost structure, possessed by cyanobacteria may allow adaptation specific to the climate change, enhancing stress tolerance [23]. Climate change may result in selection pressure for cyanobacteria, and the responses of different phenotypes of cyanobacteria to temperature stress vary [24,25]. Although the importance of external layers of cyanobacteria in the nutrient world has been recognized, direct information about the exact contribution of each type of external layer and its role in temperature stress is still very limited.

Noninvasive microtest technology (NMT) has been used in plant and algal research in recent years, and has provided new insights into net ionic and molecular fluxes by in vivo quantification [26,27]. It can truthfully reflect the flux characteristics of various ions and molecules across plant cells, due to their noninvasive advantages, as well as providing high temporal and spatial resolution, e.g., 5 s and 10 μm, respectively. In the present study, we chose two *Microcystis* species and two *Nostoc* species, which are surrounded by different types of external layers in freshwater sources. We used NMT to measure NH_4_^+^ and O_2_ net fluxes across the cyanobacterial cell surface, combined with instantaneous temperature shock to analyze their differences in tolerance. Our aims were to determine whether retention or removal of the external layers could modulate the mass transfer of cyanobacteria. The stress response to temperature of cyanobacteria was also explored. We believe that these are among the first and significant steps towards understanding the roles of various outermost layers in the adaptation and dominancy of cyanobacteria in the ever-changing environment.

## 2. Materials and Methods

### 2.1. Strains and Culturing

Two *Microcystis* species (*M. aeruginosa* FACHB-1338 and *M.* sp. FACHB-2427) and two *Nostoc* species (*N.* sp. FACHB-599 and FACHB-2009) were used in this study (Table 1). They were obtained from the Freshwater Algae Culture Collection of the Institute of Hydrobiology, Chinese Academy of Sciences (FACHB-Collection, Wuhan, China). These strains were all kept in the colony phenotype, coating with different types of external layers. The experiments were conducted in BG11 medium under a cool-white fluorescent light intensity of 50 μmol m^−2^ s^−1^ with a 12-h: 12-h light: dark cycle at a temperature of 25 ± 1 °C. The culture conditions remained the same throughout the study.

### 2.2. Experimental Design

The first phase of the study was to obtain the external-layer-retaining and stripped samples of four species. Colonial *M. aeruginosa* FACHB-1338, *M.* sp. FACHB-2427, *N.* sp. FACHB-599 and FACHB-2009 were grown in triplicate in 50-mL Erlenmeyer flasks separately. All cultures were manually shaken 3 or 4 times every day, and their position was randomized daily. Cells in the exponential growth phase in batch mode were harvested by centrifugation, and then resuspended and divided into equal halves, one for colonial samples with external layers retained, and the other for the stripped samples. To retain cell activity, *Microcystis* and *Nostoc* colonies were first centrifuged at 5000 g for 10 min, and suspended in a 0.05% NaCl solution to the original volume [7]. Low-dose ultrasound was used in our study to avoid overexposure and to achieve disaggregation of colonies and removal of the external layers [28]. *Nostoc* strains were treated by ice bath ultrasonication for 60 s at 20 kHz and 80 W (CPX-130, USA) to obtain stripped samples. *Microcystis* strains were subjected to ultrasound at 30 W for 60 s and then centrifuged at 12,000× *g* for 20 min (4 °C) to obtain stripped samples. Then, these external-layer-stripped cells were observed with India ink staining by light microscopy to check the sheath and/or slime.

In the second phase, NH_4_^+^ and O_2_ fluxes of external-layer-retaining and stripped samples of *Nostoc* and *Microcystis* were determined by NMT, to evaluate any changes after the removal of the sheath and/or slime. The external-layer-retaining and stripped samples of *Nostoc* and *Microcystis* were resuspended in BG11 N- medium, at a cell density of approximately 10^7^ cells mL^−1^, followed by culturing under routine conditions for 24 h. The photosynthetic activity of these strains was measured, and, subsequently, 1 mL culture samples were placed in the middle of poly-L-lysine-pretreated coverslips (2 × 2 cm), in a measuring chamber consisting of a glass petri dish (35 mm). After the cells had settled on the coverslips, 5 mL NMT measuring solution was gradually added to the glass petri dish. NH_4_^+^ and O_2_ fluxes of all *Nostoc* and *Microcystis* samples were then monitored by NMT, respectively.

In the third phase, to further understand the role of the sheath and/or slime in stress tolerance, an instantaneous temperature shock experiment was designed, in which *M.* sp. FACHB-2427 and *N.* sp. FACHB-2009 including two phenotypes, were used. The procedure involved the following: (i) the measuring chamber mentioned above was fixed in the center of another 100-mm glass petri dish; (ii) O_2_ fluxes were monitored at room temperature until the curve was stable; and (iii) water at different temperatures was rapidly added into the 100-mm glass petri dish separately, and an ice bath was applied at 4 °C, and a hot bath at 35 °C in the measuring chamber, respectively, with continuous O_2_ flux monitoring.

All the NMT experiments described in this section were determined by measuring at least six similar samples separately, and each measurement was repeated three times at different positions of cell.

### 2.3. Light Microscopy

External-layer-retaining and stripped samples of *Nostoc* and *Microcystis* were observed with an inverted Olympus microscope (Olympus IX73, Japan) before and after staining with India ink.

### 2.4. Photosynthetic Activity Determination

In vivo chlorophyll fluorescence was measured with a phytoplankton analyzer (PHYTO-PAM, Walz GmbH, Germany). All strains were dark-adapted for at least 15 min, before measuring the fluorescence parameters (photosystem II activity, PSII). The maximum effective quantum yield of PSII was calculated according to the following equation: *F_v_/F_m_ = (F_m_-F_0_)/F_m_* [29], where *F_m_* and *F_0_* represent the maximum and minimum fluorescence values of the dark-adapted stage of PSII, and *F_v_* is the difference between them.

### 2.5. Measurement of NH_4_^+^ and O_2_ Fluxes by NMT

Net fluxes of NH_4_^+^ and O_2_ were measured noninvasively using the noninvasive microtest technique (NMT-100 series System, Younger USA LLC, Amherst, MA, USA; Xuyue Sci. & Tech. Co., Ltd., Beijing, China). Prior to NH_4_^+^ flux determination, a prepulled and silanized microsensor (Φ1.5 ± 0.5 μm, XY-CGQ-02, Younger, USA) was first filled with a backfilling solution (100 mM NH_4_Cl) to a length of approximately 1 cm from the tip. The micropipette was front filled with 50–60 μm columns of selective liquid ion-exchange cocktails (NH_4_^+^ LIX, XY-SJ-NH4, Younger, USA). An Ag/AgCl wire microsensor holder (YG003-Y11, Younger, USA) was inserted in the back of the microsensor, to make electrical contact with the electrolyte solution. YG003-Y11 was used as the reference microsensor. For calibration of the NH_4_^+^ microsensor, we used measuring solution (0.1 mM NH_4_Cl, 0.1 mM CaCl_2_, 0.3 mM MES, 0.2 mM Na_2_SO_4_, pH 6) with different concentrations of NH_4_^+^ (0.05 mM, 0.1 mM and 0.5 mM) to choose the qualified one with a Nernstian slope at 58 ± 5 mv/decade. The microsensor was then placed in the blank measuring solution for testing by X-10 until the NH_4_^+^ flux was near the baseline, at which time the microsensor can be used [30].

To detect dissolved oxygen, the Pt/Ir polarographic oxygen microsensor (tip diameter 20 ± 5 μm, XY-CGQ-501, Younger USA) was used under -750-mV polarization voltage. A reference microsensor was also used to complete the circuit. Prior to O_2_ flux measurement, the microsensor should be calibrated with measuring solution (0.1 mM KCl, 0.1 mM CaCl_2_, 0.1 mM MgCl_2_, 0.5 mM NaCl, 0.3 mM MES, 0.2 mM Na_2_SO_4_, pH 6) containing different concentrations of O_2_ (N-saturated and control cultural media). Only when the Std Curve is between −2000~−9000 pA/mM can the microsensor be placed in the blank measuring solution for polarization for 1 h, until the net O_2_ flux is near baseline, at which time the microsensor can be used [31].

During formal measurement, the fluxes of NH_4_^+^ and O_2_ were determined by measuring six similar samples separately. The potential difference was obtained by moving the microelectrode repeatedly from one point to another, in a direction perpendicular to the surface of the individual cell (Polar X-10), and fluxes were calculated automatically by Fick’s law of diffusion: *J = −D(dc/dx)*. The steady-state flux measurements were continuously recorded for 6–10 min, and each measurement was repeated three times at different positions of the cell.

### 2.6. Data Processing and Statistical Analysis

The data obtained from NMT were exported as raw data and then converted into net fluxes by JCal V3.3 (a free MS Excel spreadsheet, http://www.youngerusa.com). For analyses of net NH_4_^+^ and O_2_ fluxes of external-layer-retaining and stripped samples of *Nostoc* and *Microcystis*, readings were averaged to obtain the net ionic and molecular steady fluxes for 6 min at each measurement position in each sample. The coefficient of variation (CV) was calculated as the standard deviation divided by the mean value. To determine the response time to instantaneous temperature shock, response curves of net O_2_ fluxes over 10 min were the first 5 points adjacent-averaging smoothed [32], and steady fluxes for 100–200 s, as appropriate, before temperature shock were averaged and regarded as the initial level. After instantaneous temperature shock, gradient recovery within ±3 pA was considered as stable data. When the smooth curve reached the initial level again, the relevant time was considered the end time of the response. Readings within the response time were integrated, and the value of integration versus time was adopted as the weighted average flux, in response to instantaneous temperature shock.

Data in this study are presented as the mean ± standard error (SE). The results of the experiment were analyzed by ANOVA, using Tukey’s post hoc test. All statistical analyses were carried out with Origin 9.0 (OriginLab, USA). Differences were considered significant at *p* < 0.05.

## 3. Results and Discussion

### 3.1. Evaluation of External Layer Extraction

**Figure 1:**
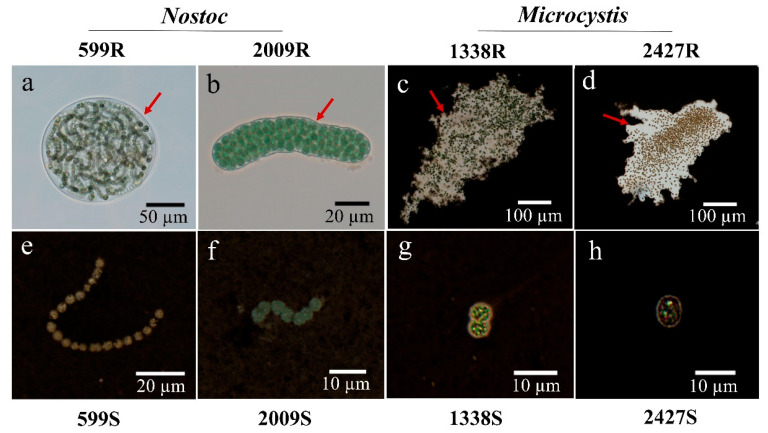
The morphology of *Nostoc* and *Microcystis* strains used in this study. (**a**,**b**) *Nostoc* embedded in sheath (arrows); (**c**,**d**) *Microcystis* embedded in slime with India ink staining (arrows); (**e**,**f**) sheath-stripped *Nostoc* in filamentous form with India ink staining; (**g**,**h**) slime-stripped *Microcystis* in unicellular form with India ink staining; R and S are the abbreviations for external-layer-retaining and stripped samples, respectively.

Optical microscopy observations showed that two strains of *Nostoc* were characterized by the presence of a firm sheath surrounding the filaments. Within the sheath, the filaments were “randomly” loosely arranged, and they showed an irregularity of coiling in *N.* sp. FACHB-599, whereas filaments in *N.* sp. FACHB-2009 were more tightly packed (Figure 1a,b). A mucilaginous layer of slime outside the colony was observed by India ink staining in two *Microcystis* strains. Multiple unicells were loosely assembled in the colony and remained irregular in shape (Figure 1c,d). After removal of the sheath and slime, the morphologies of *Nostoc* and *Microcystis* were short filaments containing 4–30 cells and single-cell forms, respectively. In these external-layer-stripped samples, neither the sheath nor the surrounding slime was observed by India ink staining, which suggests that the external layer removal was effective (Figure 1e–h).

**Figure 2:**
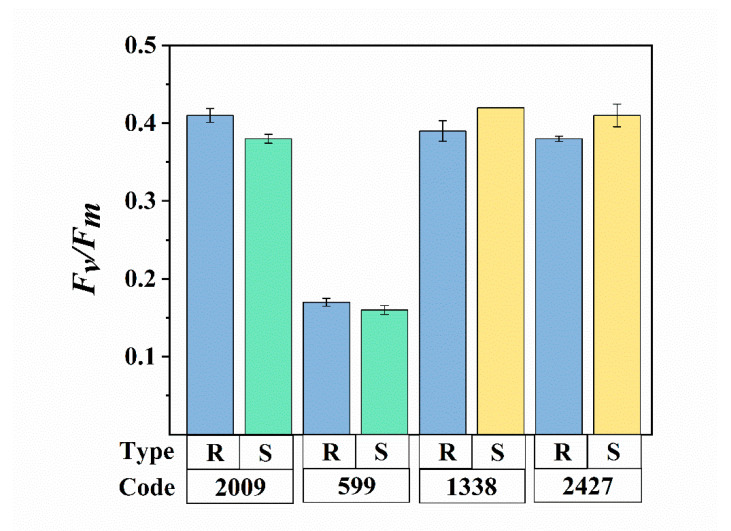
Comparison of the photosynthetic activity (*F_v_/F_m_*) between external-layer-retaining and stripped samples of *Nostoc* and *Microcystis*. R and S are the abbreviations for external-layer-retaining and stripped samples, respectively. Data are means ± SEs (*n* = 3).

The extraction methods mainly consisted of physical processes, including ultrasound and centrifugation. An excessive intensity and duration of ultrasound is known to cause rapid and severe cell disruption and photosynthesis inhibition [33]. In general, *F_v_/F_m_* is used as a sensitive indicator of photosynthetic performance, in response to environmental stress [34]. The different *F_v_/F_m_* values between species reflect their distinct differences in the potential quantum efficiency of PSII. Changes in *F_v_/F_m_*, resulting from ultrasound assisted extraction, are shown in Figure 2. In *N.* sp. FACHB-599 and FACHB-2009, the ratio of *F_v_/F_m_* deceased slightly in the sheathless strains, compared with the colonial ones, while the *F_v_/F_m_* value increased in the slimeless strains of *M. aeruginosa* FACHB-1338 and *M.* sp. FACHB-2427. However, no significant difference was observed in the *F_v_/F_m_* value between all the external-layer-retaining and stripped strains (ANOVA, *p* > 0.05), indicating that the low-frequency and power of the ultrasound applied in our study did not induce physiological deactivation of *Nostoc* and *Microcystis*. This result is consistent with the findings of Francko et al. [35], who concluded that low-dose ultrasound (50 W, 20 kHz) could provide an environmentally safe method for enhancing cyanobacterial growth. In contrast, Zhang et al. [36] reported that sonication effectively damaged cyanobacterial photosynthesis. We speculate that these discrepancies might be due to the variation in the ultrasonic conditions employed, and different morphologies of species. Purcell et al. [37] proposed that the susceptibility of microalgae to ultrasound may vary, depending on the morphological differences in shape and cell wall structure. In our experiments, the presence of different external layers was also a probable reason for various physiological changes. Nevertheless, the above results supported our assumptions that these ultrasonic treatments did not cause a significant eco-physiological change in our cyanobacteria samples, providing the basis for follow-up analysis and discussions.

### 3.2. Comparison of Net NH_4_^+^ and O_2_ Fluxes of External-Layer-Retaining and Stripped Samples

**Figure 3:**
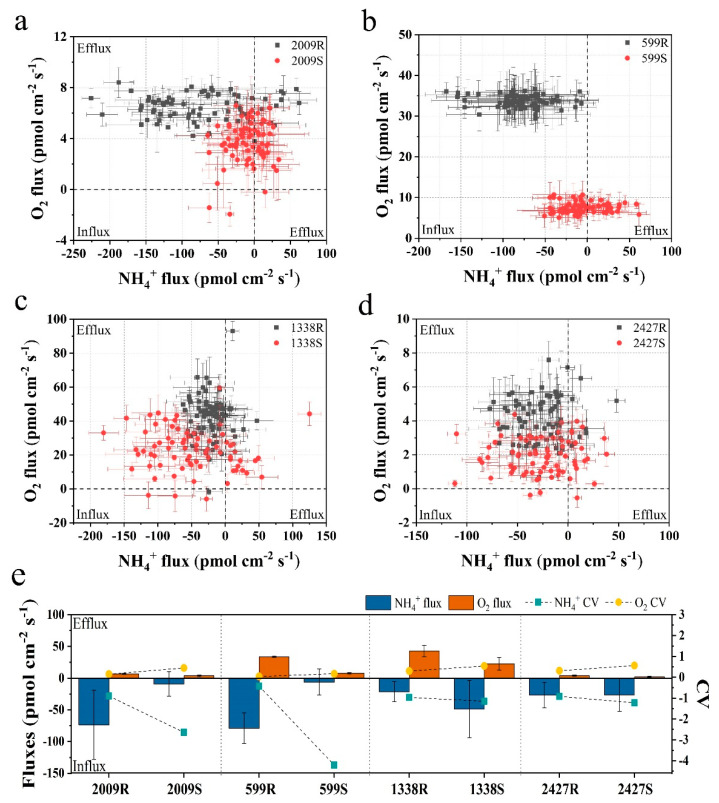
Scatter diagram and the average of NH_4_^+^ and O_2_ fluxes for 6 min of external-layer-retaining and stripped samples of *Nostoc* and *Microcystis* by noninvasive microtest technology (NMT). (**a**) *N.* sp. FACHB-2009; (**b**) *N.* sp. FACHB-599; (**c**) *M. aeruginosa* FACHB-1338; (**d**) *M.* sp. FACHB-2427; (**e**) the average value and coefficient of variation (CV); R and S are the abbreviations for external-layer-retaining and stripped samples, respectively. Data are means ± SEs (*n* = 6).

To understand whether external layers influence the nitrogen uptake and photosynthetic oxygen evolution of *Nostoc* and *Microcystis*, the NMT technique was employed to monitor net NH_4_^+^ and O_2_ fluxes across the cell in real-time. This technique enables the observation of ion and molecule flux characteristics of biological phenomena, wherein positive values of flux data represent efflux and negative values influx [30]. As shown in Figure 3a–d, both *Nostoc* and *Microcystis* mainly showed O_2_ effluxes in all external-layer-retaining and stripped samples, and the net O_2_ efflux in the retaining sample was significantly higher than its corresponding stripped sample (ANOVA, *p* < 0.05). The average net O_2_ effluxes of strains with external layers in *N.* sp. FACHB-599 and FACHB-2009, *M. aeruginosa* FACHB-1338, and *M.* sp. FACHB-2427 were 4.45-fold, 1.74-fold, 1.88-fold and 2.14-fold higher than those of the corresponding stripped samples, respectively (Figure 3e). Our findings are in accord with Wu and Song [38], who found that *Microcystis* in the colonial form has higher photosynthetic rates per unit chlorophyll compared with the unicellular form. Caution must be observed in making inferences from our results, because of the changes in size and morphology of cyanobacteria after external layer extraction. It has been generally accepted that photosynthetic parameters and nutrient uptake are related to the phenotype of cyanobacteria [38,39]. However, in the present study, the NMT platform only allowed the positioning of microelectrodes at a point near a single cell, regardless of the phenotype, e.g., colonial, filamentous, or unicellular forms. Under such an experimental microenvironment, differences in net NH_4_^+^ and O_2_ fluxes among the phenotypes with varying size were not significant (Table S1). The readily visible difference between unicellular/filamentous and colonial forms in our NMT microenvironment was the absence in external layers of the former. Thus, mass transfer of retaining and stripped samples of cyanobacteria at the submarginal level were comparable in our NMT experiments. We believe that the existence of external layers is an important feature driving O_2_ efflux in cyanobacteria.

Regarding net NH_4_^+^ flux, the two sheathed *Nostoc* strains showed obvious NH_4_^+^ influx, while the net NH_4_^+^ fluxes of corresponding sheathless strains fluctuated approximately between −50 and 50 pmol cm^−2^ s^−1^, which was significantly lower than the sheathed strain (ANOVA, *p* < 0.05) (Figure 3a,b). The average net NH_4_^+^ influxes of sheathed strains in *N.* sp. FACHB-599 and FACHB-2009 were 12.82-fold and 8.21-fold higher than the corresponding sheathless strain, respectively (Figure 3e). However, after slime removal, the net NH_4_^+^ influx in *M. aeruginosa* FACHB-1338 was higher, with an average value that was 2.29× its slime strain. No significant differences in the net NH_4_^+^ flux were observed between the slime and slimeless strains of *M.* sp. FACHB-2427 (ANOVA, *p* > 0.05) (Figure 3c–e). Moreover, the CV values of the net NH_4_^+^ and O_2_ fluxes of external-layer-retaining strains were significantly lower when compared with the corresponding stripped strain in both *Nostoc* and *Microcystis* (ANOVA, *p* < 0.05), indicating that the external-layer-retaining strains showed little fluctuation and more stable NH_4_^+^ and O_2_ fluxes (Figure 3e). These findings, again, supported the important role of external layers in cyanobacteria in ion and molecule fluxes, whereas such effects may vary, depending on the morphotype of the external layers. One of the functions of external layers in cyanobacteria may facilitate their absorption of essential nutrients that are present in the surrounding medium at submarginal levels [21]. Unlike *Nostoc* colonies with a sheath form covered by a fibrillar structure, the colonial *Microcystis* exhibits a thick mucilaginous matrix, displaying a diffuse and loosely bound structure [12], leading to the apparent differences in the diffusion boundary layer associated with nutrient transport at the cell surface [40]. The poor contribution of slime to NH_4_^+^ influx in *Microcystis* in our study was probably due to the decreased diffusive conductance of the boundary layer around colonies, compared with isolated slimeless unicells [41]. Thus, the effective diffusivity and storage of NH_4_^+^ may not be a significant feature of the slime surrounding *Microcystis* in comparison to *Nostoc*.

### 3.3. The Responses of External-Layer-Retaining and Stripped Samples to Instantaneous Temperature Shock

**Figure 4:**
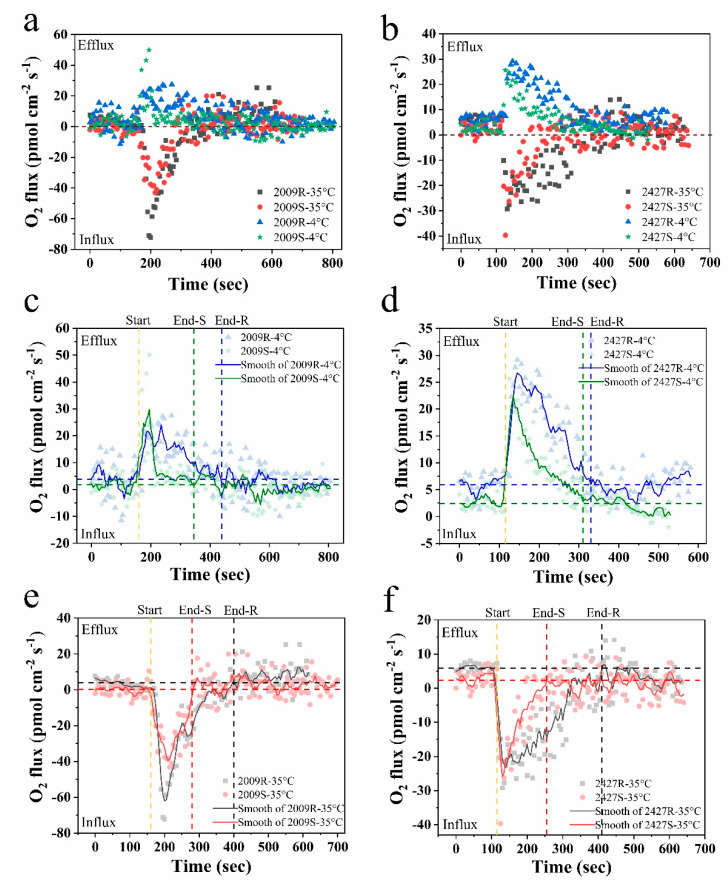
The time courses of net O_2_ flux of external-layer-retaining and stripped samples of *Nostoc* and *Microcystis* under instantaneous temperature shock at 4 °C and 35 °C by NMT, respectively. (**a**,**c**,**e**) *N.* sp. FACHB-2009; (**b**,**d**,**f**) *M.* sp. FACHB-2427; (**c**–**f**) the smoothed response curves of net O_2_ flux by the adjacent-averaging method; start, onset time of temperature shock; end, time to return to initial state; R and S are the abbreviations for external-layer-retaining and stripped samples, respectively; horizontal blue and black dotted lines are the average level of the initial state of retaining samples; horizontal green and red dotted lines are the average level of the initial state of stripped samples.

Cyanobacterial external layers, which are mainly composed of complex heteropolysaccharides, play an important physiological role in bloom formation and various types of stress tolerance during adverse conditions [9]. In this study, the real-time net O_2_ flux in *N.* sp. FACHB-2009 and *M.* sp. FACHB-2427 in external-layer-retaining and stripped samples were detected under instantaneous temperature shock at 4 °C and 35 °C, respectively (Figure 4a,b). The results showed that both retaining and stripped samples of *Nostoc* and *Microcystis* displayed remarkable O_2_ effluxes at 4 °C shock and O_2_ influxes at 35 °C, indicating a significant stimulation of respiration by 35 °C compared with 4 °C shock. We infer that this difference is due to the tendency of respiration to be more sensitive to temperature and to increase more than photosynthesis [42]. We then smoothed the response curves of net O_2_ fluxes over 10 min using the five point adjacent-averaging method. The temperature responses of the net O_2_ flux in all strains were unimodal, with rates rising up to a peak and declining thereafter at 4 °C shock (Figure 4c,d), whereas the complete reverse trend was observed at 35 °C shock (Figure 4e,f). Eventually, they all gradually returned to the initial state, which suggests that *Nostoc* and *Microcystis* have a temperature fluctuation tolerance over short time scales.

Based on the smoothed response curves of net O_2_ flux, the response time to instantaneous temperature shock of external-layer-retaining strains was significantly longer than the stripped strains at both 4 °C and 35 °C (ANOVA, *p* < 0.05) (Figure 4c–f). The retaining strains of *N.* sp. FACHB-2009 and *M.* sp. FACHB-2427 had a response time of 280, 215 s to 4 °C shock and 240 s and 295 s to 35 °C shock, whereas the stripped strains had values of 185 s, 195 s and 120 s, 140 s, respectively (Table 2), suggesting that the external layers may provide a buffer function under instantaneous temperature shock. Concomitantly, the mass flow of O_2_ within the response time increased significantly in the retaining strains at both 4 °C and 35 °C (ANOVA, *p* < 0.05). The weighted average flux within the response time are shown in Table 2. The retaining strains of *N.* sp. FACHB-2009 and *M.* sp. FACHB-2427 showed greater net O_2_ effluxes than the stripped strains at 4 °C shock (ANOVA, *p* < 0.05), probably because retaining strains can have a higher photosynthetic rate during cold adaptation. Compared with the sheathless strain of *N.* sp. FACHB-2009, the net O_2_ influx in the sheath strain was significantly lower at 35 °C shock. However, for *M.* sp. FACHB-2427 at 35 °C shock, the net O_2_ influx in the slime strain was slightly lower than the slimeless strain, although this difference was not significant (ANOVA, *p* > 0.05). Crucially, the respiration rate of phytoplankton rises more rapidly with increased temperature than the photosynthetic rate, resulting in universal declines in the rate of carbon fixation with short-term increases in temperature [43,44]. Through a comparative analysis of experimental data, it could be considered that cyanobacteria embedded in external layers have the advantage in thermal adaptation via downregulation of the respiration rate, thereby increasing the potential for carbon allocation to growth [42]. The external layers are fundamental to this adaptability especially in soil crust cyanobacteria. Previous work has indicated that some crust-forming cyanobacteria increased EPS secretion when subjected to diurnal temperature cycles [45]. This could also help explain why desert cyanobacteria, such as EPS-rich *Nostoc* colonies, can grow well when undergoing large temperature fluctuations on a daily basis [46]. Our findings suggest that external layers, especially the dense sheath, may therefore have an ameliorating impact on the efficiency of photosynthesis and photosynthesis-coupled respiration in cyanobacteria when suffering short-term temperature fluctuation.

### 3.4. Contribution of External Layers to the Dominance of Cyanobacteria

Increasing concern about cyanobacterial blooms worldwide has motivated research on their external layers and EPS secretion [9]. Cyanobacteria possess a variety of competitive advantages against their opponents, which allows them to be persistently dominant in freshwaters, most of which are considered to be related to the external gel-like matrix surrounding the cells. For instance, a coating of extracellular polysaccharidic material is involved in regulating buoyancy, chelating necessary metal cations, blocking chemical contaminants, and resisting turbulence [14,47,48]. Many studies, in fact, have shown that cyanobacterial external layers or bound EPS are the main contributors to colony formation, which would influence the development of cyanobacterial blooms [49]. Xu et al. [7] also found that stripped and removed bound EPS decreased cohesion and aggregation abilities by changing the surface properties of cyanobacterial cells, leading to the destabilization of cyanobacteria in water. Therefore, understanding the behavioral characteristics and functions of cyanobacterial external layers could be key to preventing bloom formation.

Typically, the presence or absence of an external gelatinous layer is considered an important adaptation mechanism of cyanobacteria to their environment [50]. As previously reported, the addition of minute increments of inorganic nutrients may have substantial effects on the growth of cyanobacteria in natural waters, but it has no effect in laboratory media, because the gelatinous layer is usually quickly lost in laboratory strains [21]. Our results further support and explain how the presence of external layers influence mass transfer in cyanobacteria. On the one hand, cyanobacteria cells are able to actively take up nutrients from the boundary layer adjacent to the cell, and the presence of a gelatinous coat offers an effective way to increase the prospects of encountering nutrient molecules in water [9]. On the other hand, gelatinous layers can simultaneously maintain a unique microenvironment, which allows cells to rapidly take up nutrients across the cell wall. This process creates an immediate environment, in which the nutrient concentration is more dilute than in the medium, further contributing to a beneficial inward diffusion gradient of nutrients from the medium to the gelatinous layers [13]. However, it is also apparent that the investment of external gelatinous layers is taxon-specific, and the thickness and texture of the layers are different, and the environmental response is varied [14]. Thus, it is possible that provision of a mucilaginous coat of slime offers a different balance in the intracellular proportions of carbon, nitrogen, and phosphorus, when compared to the gelatinous coat sheath. These proportions further conspicuously influence carbon or nutrient cycling in water environments [51].

With global climate changes, cyanobacterial blooms are predicted to expand. External layers of cyanobacteria can also function as a self-defense mechanism, to protect cells from climate changes. As proposed by Reynolds [13], external layer production originated as a mechanism for regulating the accumulation of photosynthate in cells, which is not released in solution. Margalef [52] observed that the sheath coating the cell can minimize unnecessary metabolic activity by slowing down diffusion. In this sense, it is possible to infer that the creation of a gelatinous layer around the cells might facilitate the regulation of mass uptake and loss, mediate interplay between photosynthesis and photosynthesis-coupled respiration, and stabilize cell activity during periods of temperature fluctuation. Overall, the characterization and function of external layers are essential to gathering information about the persistent dominance of cyanobacteria in freshwaters. Clearly, further work will be required to understand such adaptive benefits and mechanisms.

## 4. Conclusions

In conclusion, the external layer extraction methods employed in the present study can provide stripped strains, and had no statistical influence on their photosynthetic activity. Through NMT analysis, we show that external-layer-retaining strains have higher net O_2_ effluxes than stripped strains, while the role of slime in NH_4_^+^ absorption is limited compared with that of sheath. Our instantaneous temperature shock experiments suggested that external-layer-retaining strains have temperature fluctuation tolerance over short time scales. We also deduced that the external layers, especially the dense sheath, are essential for this adaptation, and may have a buffer function and ameliorating impact on the efficiency of photosynthesis and photosynthesis-coupled respiration. These findings provide key insights into the dominance of cyanobacteria during climate changes.

## Figures and Tables

**Table 1:** Characteristics of the *Nostoc* and *Microcystis* strains used in this study.

Strains	Code	Size	Form	External Layer Types
*Nostoc* sp.	FACHB-599	100–600 μm	Colony	Sheath
*Nostoc* sp.	FACHB-2009	100–600 μm	Colony	Sheath
*Microcystis aeruginosa*	FACHB-1338	200–500 μm	Colony	Slime
*Microcystis* sp.	FACHB-2427	200–500 μm	Colony	Slime

**Table 2:** Parameters of net O_2_ flux curves of *Nostoc* and *Microcystis* strains in response to instantaneous temperature shock by NMT. T_r_, response time to instantaneous temperature shock (s); M_a_, the mass flow of O_2_ within the response time (pmol cm^−2^); Fl_w_, the weighted average flux within the response time (pmol cm^−2^ s^−1^); R and S are the abbreviations for external-layer-retaining and stripped samples, respectively.

Strains	Code	T_r_		M_a_		Fl_w_	
		4 °C	35 °C	4 °C	35 °C	4 °C	35 °C
*Nostoc* sp.	2009R	280	240	3650.25	−4317.74	13.04	−17.99
	2009S	185	120	1758.13	−2951.77	9.50	−24.60
*Microcystis* sp.	2427R	215	295	3650.11	−2898.03	16.98	−9.82
	2427S	195	140	2052.27	−1386.07	10.52	−9.90

## Supplementary Materials

The following are available online at https://www.mdpi.com/2076-2607/8/6/861/s1, Table S1: Spearman correlation analysis of cyanobacterial size and fluxes (including NH_4_^+^ and O_2_ fluxes) by NMT in the experiment (n = 12).

## References

[1] Bennett H.S. (1963). Morphological aspects of extracellular polysaccharides. J. Histochem. Cytochem..

[2] Leak L.V. (1967). Fine structure of the mucilaginous sheath of *Anabaena* sp.. J. Ultrastruct. Res..

[3] Bayer M.E., Thurrow H. (1977). Polysaccharide capsule of *Escherichia coli*: Microscope study of its size, structure, and sites of synthesis. J. Bacteriol..

[4] Walsby A.E. (1968). Mucilage secretion and the movements of blue-green algae. Protoplasma.

[5] Plude J.L., Parker D.L., Schommer O.J., Timmerman R.J., Hagstrom S.A., Joers J.M., Hnasko R. (1991). Chemical characterization of polysaccharide from the slime layer of the cyanobacterium *Microcystis flos-aquae* C3-40. Appl. Environ. Microb..

[6] Otero A., Vincenzini M. (2003). Extracellular polysaccharide synthesis by *Nostoc* strains as affected by N source and light intensity. J. Biotechnol..

[7] Xu H., Jiang H., Yu G., Yang L. (2014). Towards understanding the role of extracellular polymeric substances in cyanobacterial *Microcystis* aggregation and mucilaginous bloom formation. Chemosphere.

[8] Tan X., Shu X., Duan Z., Parajuli K. (2020). Two types of bound extracellular polysaccharides and their roles in shaping the size and tightness of *Microcystis* colonies. J. Appl. Phycol..

[9] Kumar D., Kaštánek P., Adhikary S.P. (2018). Exopolysaccharides from cyanobacteria and microalgae and their commercial application. Curr. Sci..

[10] Delattre C., Pierre G., Laroche C., Michaud P. (2016). Production, extraction and characterization of microalgal and cyanobacterial exopolysaccharides. Biotechnol. Adv..

[11] Rossi F., De Philippis R., Borowitzka M., Beardall J., Raven J. (2016). Exocellular polysaccharides in microalgae and cyanobacteria: Chemical features, role and enzymes and genes involved in their biosynthesis. The Physiology of Microalgae. Developments in Applied Phycology.

[12] De Philippis R., Vincenzini M. (1998). Exocellular polysaccharides from cyanobacteria and their possible applications. FEMS Microbiol. Rev..

[13] Reynolds C.S. (2007). Variability in the provision and function of mucilage in phytoplankton: Facultative responses to the environment. Hydrobiology.

[14] Pereira S., Zille A., Micheletti E., Moradas-Ferreira P., De Philippis R., Tamagnini P. (2009). Complexity of cyanobacterial exopolysaccharides: Composition, structures, inducing factors and putative genes involved in their biosynthesis and assembly. FEMS Microbiol. Rev..

[15] Liu L.Z., Huang Q., Qin B.Q. (2018). Characteristics and roles of *Microcystis* extracellular polymeric substances (EPS) in cyanobacterial blooms: A short review. J. Freshw. Ecol..

[16] Burford M.A., Carey C.C., Hamilton D.P., Huisman J., Paerl H.W., Wood S.A., Wulff A. (2020). Perspective: Advancing the research agenda for improving understanding of cyanobacteria in a future of global change. Harmful Algae.

[17] Gan N., Xiao Y., Zhu L., Wu Z., Liu J., Hu C., Song L. (2012). The role of microcystins in maintaining colonies of bloom-forming *Microcystis* spp.. Environ. Microbiol..

[18] Reynolds C.S. (2006). The Ecology of Phytoplankton.

[19] Xiao M., Li M., Reynolds C.S. (2018). Colony formation in the cyanobacterium *Microcystis*. Biol. Rev..

[20] Fang F., Yang L., Gan L., Guo L., Hu Z., Yuan S., Chen Q., Jiang L. (2014). DO, pH, and Eh microprofiles in cyanobacterial granules from Lake Taihu under different environmental conditions. J. Appl. Phycol..

[21] Lange W. (1976). Speculations on a possible essential function of the gelatinous sheath of blue-green algae. Can. J. Micobiol..

[22] Hou J., Yang Y., Wang P., Wang C., Miao L., Wang X., Lv B., You G., Liu Z. (2017). Effects of CeO_2_, CuO, and ZnO nanoparticles on physiological features of *Microcystis aeruginosa* and the production and composition of extracellular polymeric substances. Environ. Sci. Pollut. Res..

[23] Carey C.C., Ibelings B.W., Hoffmann E.P., Hamilton D.P., Brookes J.D. (2012). Eco-physiological adaptations that favour freshwater cyanobacteria in a changing climate. Water Res..

[24] Duan Z., Tan X., Parajuli K., Upadhyay S., Zhang D., Shu X., Liu Q. (2018). Colony formation in two *Microcystis* morphotypes: Effects of temperature and nutrient availability. Harmful Algae.

[25] Griffith A.W., Gobler C.J. (2020). Harmful algal blooms: A climate change co-stressor in marine and freshwater ecosystems. Harmful Algae.

[26] Shabala S., Bose J., Volkov A.G. (2012). Application of non-invasive microelectrode flux measurements in plant stress physiology. Plant Electrophysiology.

[27] Chen H., Zhang Y.M., He C.L., Wang Q. (2014). Ca^2+^ signal transduction related to neutral lipid synthesis in an oil-producing green alga *Chlorella* sp. C2. Plant Cell Physiol..

[28] Strieth D., Stiefelmaier J., Wrabl B., Schwing J., Schmeckebier A., Nonno S.D., Muffler K., Ulber R. (2020). A new strategy for a combined isolation of EPS and pigments from cyanobacteria. J. Appl. Phycol..

[29] Maxwell K., Johnson G.N. (2000). Chlorophyll fluorescence—A practical guide. J. Exp. Bot..

[30] Zhang C., Meng S., Li Y., Zhao Z. (2014). Net NH_4_^+^ and NO_3_^−^ fluxes, and expression of NH_4_^+^ and NO_3_^−^ transporter genes in roots of *Populus simonii* after acclimation to moderate salinity. Trees.

[31] Li H.B., Zheng X.W., Tao L.X., Yang Y.J., Gao L., Xiong J. (2019). Aeration increases cadmium (Cd) retention by enhancing iron plaque formation and regulating pectin synthesis in the roots of rice (*Oryza sativa*) seedlings. Rice.

[32] Fellows A.P., Casford M.T.L., Davies P.B. (2020). Spectral Analysis and Deconvolution of the Amide I Band of Proteins Presenting with High-Frequency Noise and Baseline Shifts. Appl. Spectrosc..

[33] Tan X., Shu X., Guo J., Parajuli K., Zhang X., Duan Z. (2018). Effects of low-frequency ultrasound on *Microcystis aeruginosa* from cell Inactivation to disruption. Bull. Environ. Contam. Toxicol..

[34] Beecraft L., Watson S.B., Smith R.E.H. (2019). Innate resistance of PSII efficiency to sunlight stress is not an advantage for cyanobacteria compared to eukaryotic phytoplankton. Aquat. Ecol..

[35] Francko D., Taylor S.R., Thomas B.J., McIntosh D. (1990). Effect of low-dose ultrasonic treatment on physiological variables in *Anabaena flos-aquae* and *Selenastrum capricornutum*. Biotechnol. Lett..

[36] Zhang G., Zhang P., Liu H., Wang B. (2006). Ultrasonic damages on cyanobacterial photosynthesis. Ultrason. Sonochem..

[37] Purcell D., Parsons S.A., Jefferson B. (2013). The influence of ultrasound frequency and power, on the algal species *Microcystis aeruginosa*, *Aphanizomenon flos-aquae*, *Scenedesmus subspicatus* and *Melosira* sp.. Environ. Technol..

[38] Wu Z., Song L. (2008). Physiological comparison between colonial and unicellular forms of *Microcystis aeruginosa* Kütz. (Cyanobacteria). Phycologia.

[39] Marañón E. (2015). Cell size as a key determinant of phytoplankton metabolism and community structure. Annu. Rev. Mar. Sci..

[40] Sand-Jensen K. (2014). Ecophysiology of gelatinous *Nostoc* colonies: Unprecedented slow growth and survival in resource-poor and harsh environments. Ann. Bot..

[41] Beardall J., Allen D., Bragg J., Finkel Z.V., Flynn K.J., Quigg A., Rees T.A.V., Richardson A., Raven J.A. (2009). Allometry and stoichiometry of unicellular, colonial and multicellular phytoplankton. New Phytol..

[42] Padfield D., Yvon-Durocher G., Buckling A., Jennings S., Yvon-Durocher G. (2016). Rapid evolution of metabolic traits explains thermal adaptation in phytoplankton. Ecol. Lett..

[43] Schaum C.-E., Barton S., Bestion E., Buckling A., Garcia-Carreras B., Lopez P., Lowe C., Pawar S., Smirnoff N., Trimmer M. (2017). Adaptation of phytoplankton to a decade of experimental warming linked to increased photosynthesis. Nat. Ecol. Evol..

[44] Barton S., Jenkins J., Buckling A., Schaum C.-E., Smirnoff N., Raven J.A., Yvon-Durocher G. (2020). Evolutionary temperature compensation of carbon fixation in marine phytoplankton. Ecol. Lett..

[45] Wang W., Wang Y., Shu X., Zhang Q. (2013). Physiological responses of soil crust-forming cyanobacteria to diurnal temperature variation. J. Basic Microbiol..

[46] Schmidt S.K., Vimercati L. (2019). Growth of cyanobacterial soil crusts during diurnal freeze-thaw cycles. J. Microbiol..

[47] Li M., Xiao M., Zhang P., Hamilton D.P. (2018). Morphospecies-dependent disaggregation of colonies of the cyanobacterium *Microcystis* under high turbulent mixing. Water Res..

[48] Chen M., Tian L., Ren C., Xu C., Wang Y., Li L. (2019). Extracellular polysaccharide synthesis in a bloom-forming strain of *Microcystis aeruginosa*: Implications for colonization and buoyancy. Sci. Rep..

[49] Kaplan Can H., Gurbuz F., Odabaşı M. (2019). Partial characterization of cyanobacterial extracellular polymeric substances for aquatic ecosystems. Aquat. Ecol..

[50] Borah D., Nainamalai S., Gopalakrishnan S., Rout J., Alharbi N.S., Alharbi S.A., Nooruddin T. (2018). Biolubricant potential of exopolysaccharides from the cyanobacterium *Cyanothece epiphytica*. Appl. Microbiol. Biot..

[51] Pannard A., Pedrono J., Bormans M., Briand E., Claquin P., Lagadeuc Y. (2016). Production of exopolymers (EPS) by cyanobacteria: Impact on the carbon-to-nutrient ratio of the particulate organic matter. Aquat. Ecol..

[52] Margalef R. (1997). Our Biosphere.

