# Supplementary material for: The Role of Cyanobacterial External Layers in Mass Transfer: Evidence from Temperature Shock Experiments by Noninvasive Microtest Technology
Source: Microorganisms. 2020 Jun 7;8(6):861. doi: 10.3390/microorganisms8060861 (PMC7356193; doi:10.3390/microorganisms8060861)
Supplement: Supplementary file 1 [file microorganisms-08-00861-s001.pdf]

Supplementary material

The role of cyanobacterial external layers in mass transfer: evidence from temperature shock experiments by noninvasive microtest technology

Yan Xiao, Lingxin Liu, Zhe Li*, Yuran Cheng

CAS Key Laboratory on Reservoir Water Environment, Chongqing Institute of Green and Intelligent Technology, Chinese Academy of Sciences
No. 266 Fangzheng Avenue, Shuitu Hi-tech Industrial Park, Shuitu Town, Beibei District, Chongqing 400714, China

Corresponding author: Zhe Li
Email: lizhe@cigit.ac.cn
Tel.: +86-23-65935092
Fax: +86-23-65935000

Table S1. Spearman correlation analysis of cyanobacterial size and fluxes (including NH_4^+ and O_2 fluxes) by noninvasive microtest technology (NMT) in the experiment (n = 12).

Strains	Code	NH_4^+ flux		O_2 flux	
		correlation coefficient	*P* value	correlation coefficient	*P* value
***Nostoc* sp.**	**FACHB-2009**	-0.527	0.179	0.487	0.268
***Nostoc* sp.**	**FACHB-599**	-0.667	0.071	0.783	0.118
Microcystis aeruginosa	**FACHB-1338**	0.360	0.427	0.791	0.111
***Microcystis* sp.**	**FACHB-2427**	0.168	0.643	0.564	0.322
